# Comparative analysis of combined phosphorus and drought stress-responses in two winter wheat

**DOI:** 10.7717/peerj.13887

**Published:** 2022-09-22

**Authors:** Xiangchi Zhang, Chao Li, Weidan Lu, Xiaoli Wang, Bin Ma, Kaiyong Fu, Chunyan Li, Cheng Li

**Affiliations:** Shihezi University, Shihezi, China

**Keywords:** Drought stress, Phosphorus, Root, Wheat, Abiotic stress

## Abstract

Phosphorus stress and drought stress are common abiotic stresses for wheat. In this study, two winter wheat varieties “Xindong20” and “Xindong23” were cultured in a hydroponic system using Hoagland nutrient solution and treated with drought stress under conventional (CP: 1.0 mmol/L) and low (LP: 0.05 mmol/L) phosphorus levels. Under drought stress, the root growth was better under LP than under CP. Under LP, root phosphorus content was increased by 94.2% in Xindong20 and decreased by 48.9% in Xindong23 at 3 d after re-watering, compared with those at 0 d under drought stress. However, the potassium (K) content was the highest among the four elements studied and the phosphorus (P) and calcium (Ca) content were reduced in the root of the two varieties. Under CP, the zinc (Zn) content was higher than that under LP in Xindong23. The GeneChip analysis showed that a total of 4,577 and 202 differentially expressed genes (DEGs) were detected from the roots of Xindong20 and Xindong23, respectively. Among them, 89.9% of DEGs were involved in organelles and vesicles in Xindong20, and 69.8% were involved in root anatomical structure, respiratory chain, electron transport chain, ion transport, and enzyme activity in Xindong23. Overall, LP was superior to CP in mitigating drought stress on wheat, and the regulatory genes were also different in the two varieties. Xindong20 had higher drought tolerance for more up-regulated genes involved in the responses compared to Xindong23.

## Introduction

Wheat is one of the most important food crops in the world, and its growth is affected by many environmental factors. Phosphorus (P) is one of the essential nutrients for wheat growth. However, the distribution of soil P is uneven worldwide. Moreover, drought stress is very common in arid and semi-arid regions. Therefore, in some arid and semi-arid regions, P starvation and drought stress simultaneously threaten the growth of crops.

Under the P starvation condition, plants enhance P utilization by increasing P uptake and P utilization efficiency (Vance, Uhde-Stone & Allan, 2003). The changes in root structure, bio-availability of soil inorganic P, high-affinity P transporter, and some metabolic pathways have also been detected in plants to enhance P utilization (Liu et al., 2010). Besides, previous studies found that the mycorrhizal symbiosis is closely associated with P transporters and P utilization, and 80% of terrestrial plant increase nutrient uptake through fungal hyphae (Harrison, Dewbre & Liu, 2002; Paszkowski et al., 2002). Katrin et al. (2019) showed that P utilization could be enhanced by secreting organic acids, protons, acid phosphatases, and ribonuclease by plant roots.

Drought stress could suppress the growth of plant roots, while plants may change their morphology to adapt to the drought stress (Deak & Malamy, 2005), such long and dense roots in drought-tolerant plant (Hurd, 1975). Drought stress affects plant photosynthesis, membrane lipids, active oxygen metabolism, carbon and nitrogen metabolism, and osmotic adjustment. Previous studies showed that large quantities of reactive oxygen species were accumulated in plant roots under drought conditions, causing membrane lipid peroxidation (Moller, Jensen & Hansson, 2007; Cruz de Carvalho, 2008; Anjum et al., 2011). Therefore, maintaining high antioxidant enzyme activity is essential for enhancing plant drought tolerance (Sharma & Dubey, 2005; Türkan et al., 2005). It has been reported that the structure of the root and canopy of plants are affected by the distribution of dry matter (Hermans et al., 2006). Under drought conditions, plants produce more root branches by sucrose metabolism to enhance drought tolerance (Macgregor et al., 2008). In addition, silicon has also been proven to be one of the important elements involving in the stress tolerance of plants (Epstein, 1999; Savant et al., 1999). Silicon could affect the membrane permeability of rice under drought stress (Agarie et al., 1998). Adams, Kendall & Kartha (1990) reported that trehalose could act as a penetration protectant under drought conditions.

Water deficit affects the uptake of nutrients by crops, and the deficiency of nutrients may lead to the decrease of water use efficiency, thus affecting crop yield and quality (Manske et al., 2001; Condon et al., 2004; Wang et al., 2009). Wu et al. (2018) found that increasing nitrogen and P application could minimize the effects of drought stress on bamboo growth through increasing photosynthetic rate and membrane integrity. Root hair plays a crucial role in relieving water and P stress. Nussaume et al. (2011) found that P could be obtained by plant roots through specific transporters, such as high-affinity phosphate transporters. Besides, Kellermeier et al. (2014) showed that the nutrients in plants had a significant influence on the expression of genes that regulate root structure. For instance, Jain et al. (2013) showed that P starvation response signaling pathway and the expression of some target genes, especially Pht1;1 in the root, appear to be suppressed under zinc-deficient conditions. In ectomycorrhizal symbiosis, P and K^+^ jointly affect membrane transport and transferred to host plants (Garcia et al., 2014), while high K^+^ affects the uptake of P by *Arabidopsis* (Ródenas et al., 2019). Matthus et al. (2019) found that when P starvation occurred, *Arabidopsis* root tips showed a strong spatiotemporal [Ca^2+^] cyt response to eATP. Therefore, the change of Ca^2+^ content in plants could indicate the growth status of plant roots and the response to stress (Behera et al., 2018; Manishankar et al., 2018). However, the molecular mechanisms of plants responses to combined drought and P stress have not been reported.

High-throughput technologies, such as large-scale parallel sequencing and gene chips, have been applied in molecular biological researches on the response of model plants to P starvation. At present, many genes involved in the responses to P starvation have been identified. They play an essential role in regulating P homeostasis plants (Zhou et al., 2015). In addition, the transcriptional characteristics of wheat responses to temperature, drought, and salt stress could also be detected by microarray technology. However, at present, there are few studies on the transcriptomic responses of wheat combined phosphorus and drought stress. Therefore, in this study, the effects of different P application rates (LP and CP) on the root growth of two wheat varieties (Xinong20 and Xinong23) under drought stress were determined through the detection of root physiological information and GeneChip. This study aimed to explore the physicochemicaland transcriptomes responses of wheat roots to two P levels under drought stress. We speculated that the gene expression in wheat was changed accordingly with the P content and drought stress.

## Materials and Methods

### Plant material and cultivation

The winter wheat varieties “Xindong20” and “Xindong23” provided by the Winter Wheat Research Group of the Agricultural College of Shihezi University were used in this study. “Xindong20” has higher drought tolerance and P utilization efficiency than the “Xindong23”. To obtain complete and undamaged root systems, wheat plants were cultured in hydroponic system using Hoagland nutrient solution, in growth cabinets (JNYQ, RXZ-300B-LED, China) with an 18 h day-length under photosynthetically active light (250 µmole m^−2^ s^−1^). The temperature during the day was 25 °C and at night it was 20 °C. Plump wheat seeds were sterilized in 1‰ HgCl_2_ solution for 30 s, rinsed with distilled water, and placed evenly in a petri dish lined with wet filter paper. The petri dishes with seeds were placed in an incubator in the dark to promote germination. Distilled water was regularly replenished to keep moist. After 1 week, the seedlings with similar growth were selected and transferred to a black plastic box to maintain roots in darkness. Each box contained the same volume of Hoagland nutrient solution. The boxes were divided into conventional P level (CP: 1.0 mmol/L) and low P level (LP: 0.05 mmol/L). KCl was used to replace part of KH_2_PO_4_ under LP. The nutrient solution was changed every 3 days. After 14 d of culture, 15% (w/v) PEG-6000 (osmotic potential: −0.32 MPa) was added into the Hoagland nutrient solution (both CP and LP) to simulate drought stress on wheat seedlings for 7 d. Then the wheat seedlings were cultured with Hoagland nutrient solution without PEG-6000 under CP and LP for 3 d. The roots tissues were sampled at 0 d, 3 d, 5 d, 7 d under drought stress (DS 0 d, 3 d, 5 d, and 7 d) and 3 d after re-watering (RW 3 d). Part of the samples at DS 7 d were quickly frozen in liquid nitrogen and stored at −80 °C.

### Root morphological scanning

Fresh roots were soaked in tap water for 10 min, and then rinsed with running water. The roots were completely spread out to avoid overlapping. The plant image scanner (Wanshen LS-A, Phantom 9850XL PLUS, China) was used for scanning, and root images were stored in a computer. The root analysis system (WinRHIZO 2009 system) was used to determine the total root length, total root surfarea, and the number of forks crossings, and root tips.

### Root absorption area measurement

Surface water on fresh roots were absorbed by the filter paper. The root absorption area was determined according to the method described by Zhang (1990). Briefly, methylene blue solution (0.2 μmol/L) was transferred into three numbered small beakers. The volume of the solution in each beaker was about 10 times the volume of the root. The roots sampled at different dates were immersed in different beakers containing methylene blue solution for 1.5 min. After that 1 mL of the solution was taken from each of the three beakers and diluted 10 times. Distilled water was used for the control group. Absorbance was measured at 660 nm using a spectrophotometer (Shanghai Precision Scientific Instrument Co., Ltd., Shanghai, China).

### Determination of root vitality

The root vitality was determined using a modified method (Kang & Saltveit, 2002). Wheat root samples (0.2 g) were transferred into a 50 mL centrifuge tube, and then 5 mL of TTC solution (0.4%, w/v) and 5 mL of salt buffer (1 mol/L, pH 7.0) were added. Before incubation for 1 h at 37 °C, the roots samples were completely immersed in the reaction solution. When the root samples turned red, the methanol-soaked method was used to completely whiten the apical segments (approximately 4–6 h). Absorbance was measured at 485 nm using a spectrophotometer (Shanghai Precision Scientific Instrument Co., Ltd., Shanghai, China).

### Measurement of water content

The water content of wheat seedlings was determined by the method described by Gao (2006). Briefly, seedlings were washed under running tap water and dried using filter paper. Leaves and roots were separated, weighed, dried at 105 °C for 30 min and then tried and at 70 °C for 4–5 h to constant weight. Three replicates were adopted and ten wheat seedlings were included in each replicate.

### Measurement of total phosphorus content

The total phosphorus content was determined according to the method described by Bao (2000). Briefly, dried, and ground sample (0.2 g) was transferred in a 100 mL tube and digested by H_2_SO_4_–H_2_O_2,_ until the solution was completely clear. Then, dinitrophenol indicator and 6 M NaOH were added to neutralize the solution, and 10 mL of ammonium vanadylmolybdate reagent were added finally. Absorbance was measured at 440 nm using a spectrophotometer (Shanghai Precision Scientific Instrument Co., Ltd.722G, Shanghai, China).

### Total RNA extraction and microarray analysis of root tissue

Total RNA was extracted using a Fruit-mate (9192; Takara, Shiga, Japan) and RNAiso plus (9108; Takara, Shiga, Japan) kit, according to the manufacturer’s instructions. Then, the total RNA was quantified using NanoDrop ND-2000 (Thermo Fisher Scientific, Waltham, MA, USA), and the integrity of the RNA was detected by gel electrophoresis. The qualified samples were sent to Beijing Compass Biotechnology, China for gene chip analysis (three replicates). Sample labeling, chip hybridization, and washing were performed with reference to the standard protocol of the chip technology (3′ IVT Expression Arrays; Affymetrix, Santa Clara, CA, USA). First, the total RNA was reverse transcribed to generate a double-stranded cDNA, and the double-stranded cDNA was transcribed to generate biotin-labeled cRNA. The labeled cRNA was then fragmented and chip-hybridized. After washing and staining, the original images were obtained using an Affymetrix Scanner 3000 (7G; Affymetrix, Santa Clara, CA, USA).

Command Console software (version 4.0, Affymetrix, Santa Clara, CA, USA) was used to analyzeimages to get raw data, and then Expression Console software (version 1.4.1, Affymetrix, Santa Clara, CA, USA) was used for RMA normalization. The rationality of the experimental design and the uniformity of replicates were evaluated using principal component analysis (PCA). Then, a moderated t-test was performed to identify genes with differential expression. The *p* value 0.05 and fold change (|log2FC|) greater than one were used as the thresholds to determine the differentially expressed genes (DEGs). Additionaly, the AgriGO database (http://bioinfo.cau.edu.cn/agriGO/index.php) was used for the GO enrichment analysis of DEGs, and the Bonferroni correction method was used to adjust the *p* value to control the false discovery rate (FDR). GO items with FDR lower than 0.05 were considered significant and used to analyze the biological functions of DEGs.

### The ionome analysis

Twenty seedlings with similar growth were randomly collected from each of the CP and LP treatments. The content of calcium (Ca), potassium (K), zinc (Zn), and phosphorus (P) in root samples were determined using Agilent ICP-OES 710 (GenTech, South San Francisco, CA, USA). The element standard solution (1,000 μg/mL) was measured and diluted with 2% nitric acid to prepare the standard curve. Briefly, the oven-dried sample of 0.1 g was put in a polytetrafluoroethylene beaker, and then 10 mL of nitric acid was added. The mixture was heated on a hot plate for 12 h, and then digested at 150 °C until 2–3 mL liquid remained. After cooling, the liquid was transferred to 20 mL volumetric flask, dilute with 2% nitric acid to constant volume. Meanwhile, the blank test was done.

### qRT-PCR analysis

The rest of the wheat root samples were used for qRT-RCR reaction to verify the results of gene chip. Total RNA was extracted using the *EASY*spin Plus Complex Plant RNA Kit (RN53; AidLab, Beijing, China). The first-strand cDNA was synthesized using the *EasyScript®* One-Step gDNA Removal and cDNA Synthesis SuperMix (AE311-03; TransGen Biotech, Beijing, China). The wheat actin gene was selected as the endogenous control. According to the manufacturer’s instructions, the qRT-PCR reaction system was prepared with an SYBR Green I Master (LightCycler® 480 SYBR Green I Master; Roche, Indianapolis, IN, USA). A total of three replicates were performed in this experiment (each repetition contained 20 individuals). The primers used in this experiment were designed by Primer Premier 5 and DNAMAN software (Table 1). Each PCR reaction was repeated at least three times. Relative quantitative methods of ∆∆CT were used to analyze the changes in the number of selected genes.

**Table 1:** Primers used in qRT-PCR experiments.

Probe set ID	Forward primer (5′-3′)	Reverse primer (5′-3′)
Ta.5385.1.S1_at	TCGACAACGCCTACTACACCA	TACGTCCATCACGAGTTCACC
Ta.23044.1.A1_s_at	CGAACTCCAGGGCCACCTTC	GTCGACACCACCTCCGACAC
Ta.2758.1.S1_at	TCGTCATGTTCGGCACCATCC	CGCTCACCCTGGACGTTCTC
Ta.11120.1.S1_x_at	AACATCAACAGCACCAAGCC	TCAACAAAGCCTGCGAACGTC
Ta.5456.1.A1_at	ACCAGAGGAAGGGATTCAGTG	GTGCGAATACAATACGATGCTG

### Data analysis

The data were analyzed using Excel software and Origin software (version 9.60, OriginLab, Northampton, MA, USA). Each measurement was repeated at least three times. Significance was determined by paired t-test at *p* < 0.05. Statistical charts were drawn by using Excel software (version 16.0, Microsoft, Redmond, WA, USA) and Adobe Photoshop software CC 2019 (version 20.0, Adobe, San Jose, CA, USA).

## Results

### Effects of two phosphorus levels on morphological and physiological characteristics of wheat root under drought stress

**Figure 1:**
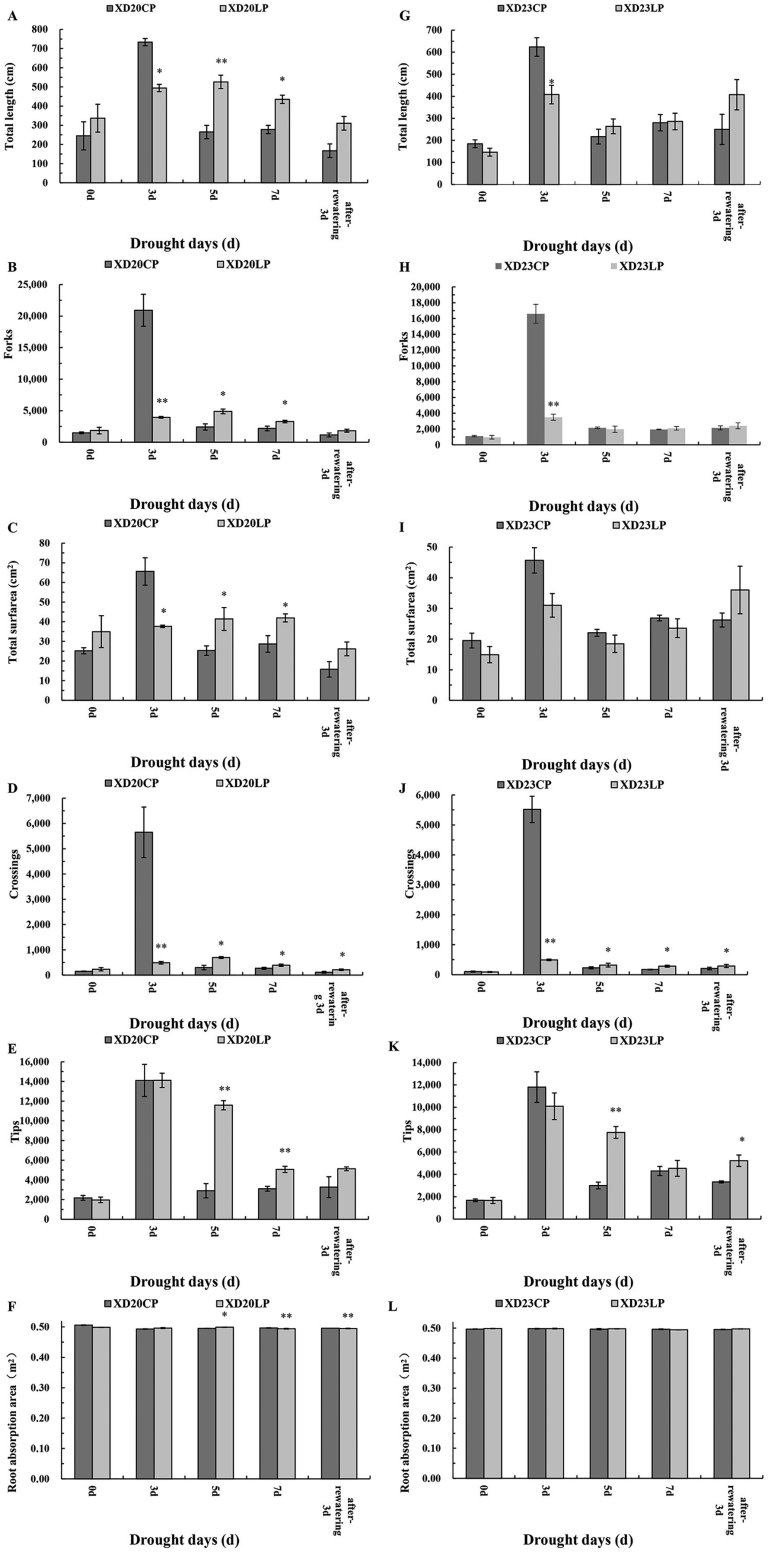
Effects of two phosphorus levels on root morphological characteristics of wheat plants at seedling stage under drought stress. CP: 1.0 mmol/L; LP: 0.05 mmol/L; Values are means ± standard deviation of three repetitions; One asterisk (*) and two asterisks (**) are significantly different at *p* < 0.05 and *p* < 0.01, respectively. XD20CP: Xindong20 under conventional phosphorus level; XD20LP: Xindong20 under low phosphorus level; XD23CP: Xindong23 under conventional phosphorus level; XD23LP: Xindong23 under low phosphorus level. (A and G) Effects of low phosphorus and conventional phosphorus levels on the total root length of two wheat varieties under drought stress. (B and H) Effects of low phosphorus and conventional phosphorus levels on the number of root forks of two wheat varieties under drought stress. (C and I) Effects of low phosphorus and conventional phosphorus levels on the root total surfarea of two wheat varieties under drought stress. (D and J) Effects of low phosphorus and conventional phosphorus levels on the number of root crossings of two wheat varieties under drought stress. (E and K) Effects of low phosphorus and conventional phosphorus levels on the number of root tips of two wheat varieties under drought stress. (F and L) Effects of low phosphorus and conventional phosphorus levels on the root absorption area of two wheat varieties under drought stress.

There were considerable morphological differences between Xindong20 and Xindong23 during drought stress both under LP and CP (Fig. 1). Because the root sampling was destructive, the variation was discontinuous. Only the differencees at the same date were considered. The root morphology changed with time in both varieties. However, compared with Xindong23, Xindong20 was more sensitive to the combined drought and phosphorus treatment. At DS 3 d, the total root length, total root surfarea, and the number of forks and crossings in Xindong20 were significantly greater under CP than under LP. However, at DS 5 d and DS 7 d, these were significantly lower under CP than under LP (Figs. 1A–1D). For Xindong23, the number of root crossings under CP was significantly different from that under LP on all sampling days except for DS 0 d (Fig. 1J). The root tips of Xindong20 was significantly more under LP than under CP at DS 5 d and DS 7 d (Fig. 1E). However, for Xindong23, the number of root tips was significantly lower under CP than under LP at DS 5 d and RW 3 d (Fig. 1K). The root absorption area of Xindong 20 was significantly greater under LP than under CP at DS 5 d, but that was significantly greater under CP than under LP at DS 7 d and RW 3 d (Fig. 1F). There was no significant difference in the root absorption area of Xindong20 between CP and LP on all sampling days (Fig. 1L).

**Figure 2:**
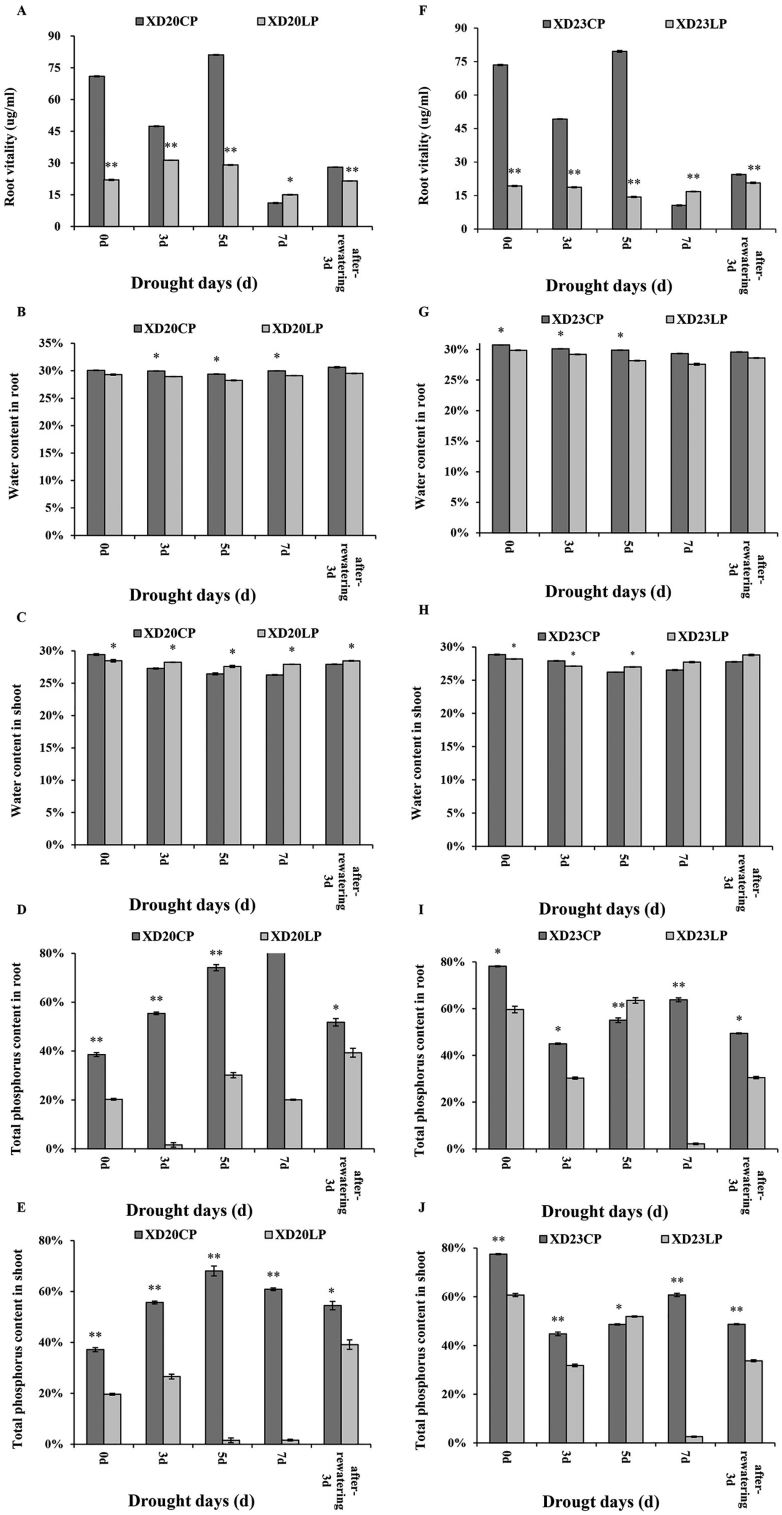
Effects of two phosphorus levels on physiological characteristics of wheat plants at seedling stage under drought stress. CP: 1.0 mmol/L; LP: 0.05 mmol/L; Values are means ± standard deviation of three repetitions; one asterisk (*) and two asterisks (**) are significantly different at *p* < 0.05 and *p* < 0.01, respectively. XD20CP: Xindong20 under conventional phosphorus level; XD20LP: Xindong20 under low phosphorus level; XD23CP: Xindong23 under conventional phosphorus level; XD23LP: Xindong23 under low phosphorus level. (A and F) Effects of low phosphorus and conventional phosphorus levels on the root vitalety of two wheat varieties under drought stress. (B and G) Effects of low phosphorus and conventional phosphorus levels on the water content in root of two wheat varieties under drought stress. (C and H) Effects of low phosphorus and conventional phosphorus levels on the water content in shoot of two wheat varieties under drought stress. (D and I) Effects of low phosphorus and conventional phosphorus levels on the total phosphorus content in root crossings of two wheat varieties under drought stress. (E and J) Effects of low phosphorus and conventional phosphorus levels on the total phosphorus content in shoot of two wheat varieties under drought stress.

The changes of root vitality in Xindong20 and Xindong23 were similar across time under CP (Figs. 2A, 2F). Under drought stress, the root vitality of both varieties were significantly higher under CP than under LP on all sampling days except for DS 7 d.

The root water content of both varieties were significantly higher under CP than under LP on all sampling days. The root water content of Xindong20 was gradually decreased from DS 3 d to DS 5 d, and then it was gradually increased (Fig. 2B). However, the root water content of Xindong23 was lowest at DS 7 d, and then gradually increased (Fig. 2G). It should be noted that under LP, the change of root water content of Xindong23 was more significant than that of Xindong20 from DS 7 d to RW 3 d. The shoot water content of Xindong20 was higher under LP than under CP from DS 3 d to RW 3 d. The changes of shoot water content in in two wheat varieties under CP were almost consistent with those under LP (Figs. 2C, 2H).

The changes of total P content in root and shoot of Xindong20 was different from those of Xindong23 (Figs. 2D, 2E, 2I and 2J). For Xindong20, from DS 0 d to RW 3 d, the total P content of root and shoot were significantly higher under CP than under LP (Figs. 2D, 2E). However, those of Xindong23 were significantly higher under CP than under LP on all sampling days except for DS 5 d (Figs. 2I, 2J).

### Identification of differential gene expression

**Figure 3:**
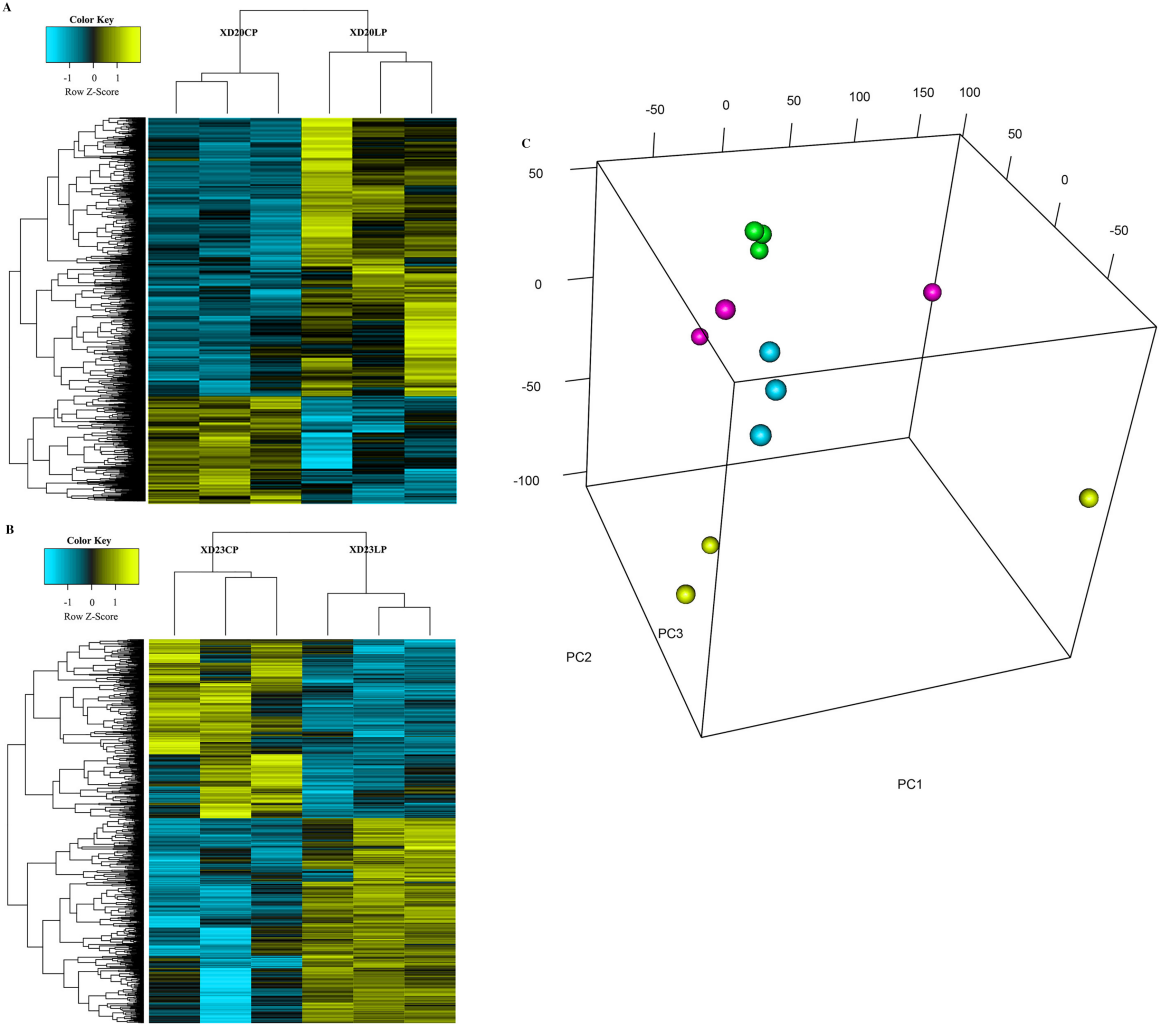
Hierarchical cluster and principal component analysis of differentially expressed genes in the root of Xindong20 and Xindong23 at 7 d drought stress under CP and LP treatments. (A) Hierarchical cluster analysis of differentially expressed genes in the root of Xindong20 at 7 d drought stress under CP and LP treatments. (B) Hierarchical cluster analysis of differentially expressed genes in the root of Xindong23 at 7 d drought stress under CP and LP treatments. (C) Principal component analysis of differentially expressed genes in the root of Xindong20 and Xindong23 at 7 d drought stress under CP and LP treatments. XD20CP: Xindong20 under conventional phosphorus level; XD20LP: Xindong20 under low phosphorus level; XD23CP: Xindong23 under conventional phosphorus level; XD23LP: Xindong23 under low phosphorus level.

**Figure 4:**
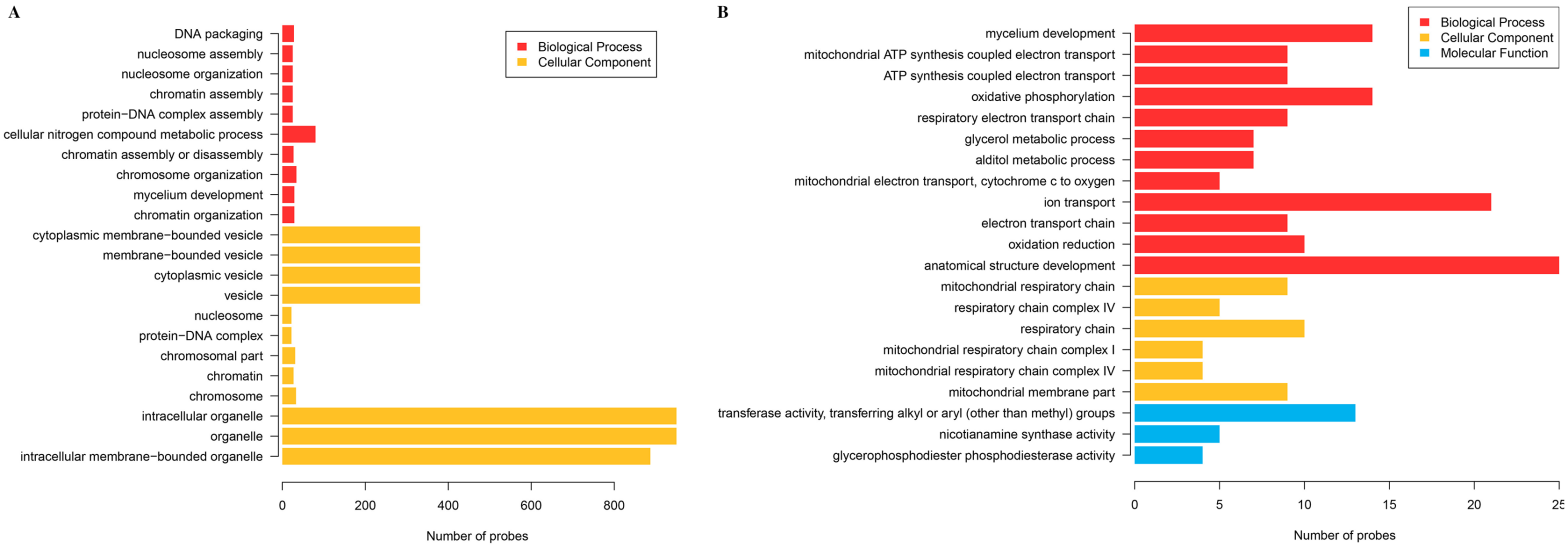
Gene Ontology analysis of differentially expressed genes in the root of Xindong20 and Xindong23 at 7 d drought stress under CP and LP treatments. (A) Gene Ontology analysis of differentially expressed genes in the root of Xindong20; (B) Gene Ontology analysis of differentially expressed genes in the root of Xindong23.

The cluster analysis and PCA of DEGs that the experimental samples were good (Fig. 3). In this study, 4,577 and 202 DEGs (*p* < 0.05 & |logFC| > 1) were detected from the roots of Xindong20 and Xindong23, respectively. Among them, 3,207 up-regulated genes and 1,370 down-regulated genes were found in Xindong20, accounting for 70.07% and 29.93% of DEGs, respectively; 55 up-regulated genes and 147 down-regulated genes were found in Xindong23, accounting for 27.23% and 72.77% of the total DEGs, respectively. The number of DEGs in Xindong20 root was 22.7 times that of Xindong23, and the number of up-regulated genes of Xindong20 was 1.3 times that of the down-regulated gene (Table 2). According to Gene Ontology (GO) enrichment analysis, the DEGs of Xindong20 were mainly divided into two categories including biological processes and cell components (Fig. 4). However, the DEGs of Xindong23 were mainly divided into three major categories including biological processes, cellular components, and molecular functions. There was a common category (regulation process-mycelium development) for the two varieties. In this category, 29 genes associated with mycelium development were all down-regulated in Xindong20. In Xindong23, among the 14 genes associated with mycelial development, were seven up-regulated and seven down-regulated (Tables 3 and 4). In addition, 13 common DEGs were found in the two varieties.

**Table 2:** Numbers of differentially expressed genes (DEGs) in two wheat genotypes. The number of differentially expressed genes were from the comparison between LP and CP treatment in the wheat root subjected to drought stress for 7 d, respectively.

Genotypes	Gene category	No. of genes	Percentage of total DEGS (%)
Xindong20	Up	3,207	70.07
	Down	1,370	29.93
Xindong23	Up	55	27.23
	Down	147	72.77

**Table 3:** The number of up-regulated and down-regulated probes in different GO terms in the root of Xindong20.

Term_type	Go-term	Up-regulated probe	Down-regulated probe
B	DNA packaging	25	3
B	Nucleosome assembly	22	3
B	Nucleosome organization	22	3
B	Chromatin assembly	22	3
B	Protein-DNA complex assembly	22	3
B	Cellular nitrogen compound metabolic process	51	29
B	Chromatin assembly or disassembly	24	3
B	Chromosome organization	29	5
B	Mycelium development	0	29
B	Chromatin organization	25	4
C	Membrane-bounded vesicle	218	114
C	Cytoplasmic membrane-bounded vesicle	218	114
C	Vesicle	218	114
C	Cytoplasmic vesicle	218	114
C	Nucleosome	19	3
C	Protein-DNA complex	19	3
C	Chromosomal part	25	6
C	Chromatin	24	3
C	Chromosome	27	6
C	Intracellular organelle	666	284
C	Organelle	666	284
C	Intracellular membrane-bounded organelle	647	240

**Note:**

B, Biological Process; C, Cellular Component.

**Table 4:** The number of up-regulated and down-regulated probes in different GO terms in the root of Xindong23.

Term_type	Go-term	Up-regulated probe	Down-regulated probe
B	Mycelium development	7	7
B	Mitochondrial ATP synthesis coupled electron transport	0	9
B	ATP synthesis coupled electron transport	0	9
B	Respiratory electron transport chain	0	9
B	Oxidative phosphorylation	3	11
B	Alditol metabolic process	7	0
B	Glycerol metabolic process	7	0
B	Mitochondrial electron transport, cytochrome c to oxygen	0	5
B	Ion transport	5	16
B	Electron transport chain	0	9
B	Oxidation reduction	1	9
B	Anatomical structure development	11	14
M	Transferase activity, transferring alkyl or aryl groups	7	6
M	Nicotianamine synthase activity	0	5
M	Glycerophosphodiester phosphodiesterase activity	4	0
C	Mitochondrial respiratory chain	1	8
C	Respiratory chain complex IV	0	5
C	Respiratory chain	1	9
C	Mitochondrial respiratory chain complex I	0	4
C	Mitochondrial respiratory chain complex IV	0	4
C	Mitochondrial membrane part	1	8

**Note:**

B, Biological Process; C, Cellular Component; M, Molecular Function.

### Functional categories of differentially expressed genes

**Figure 5:**
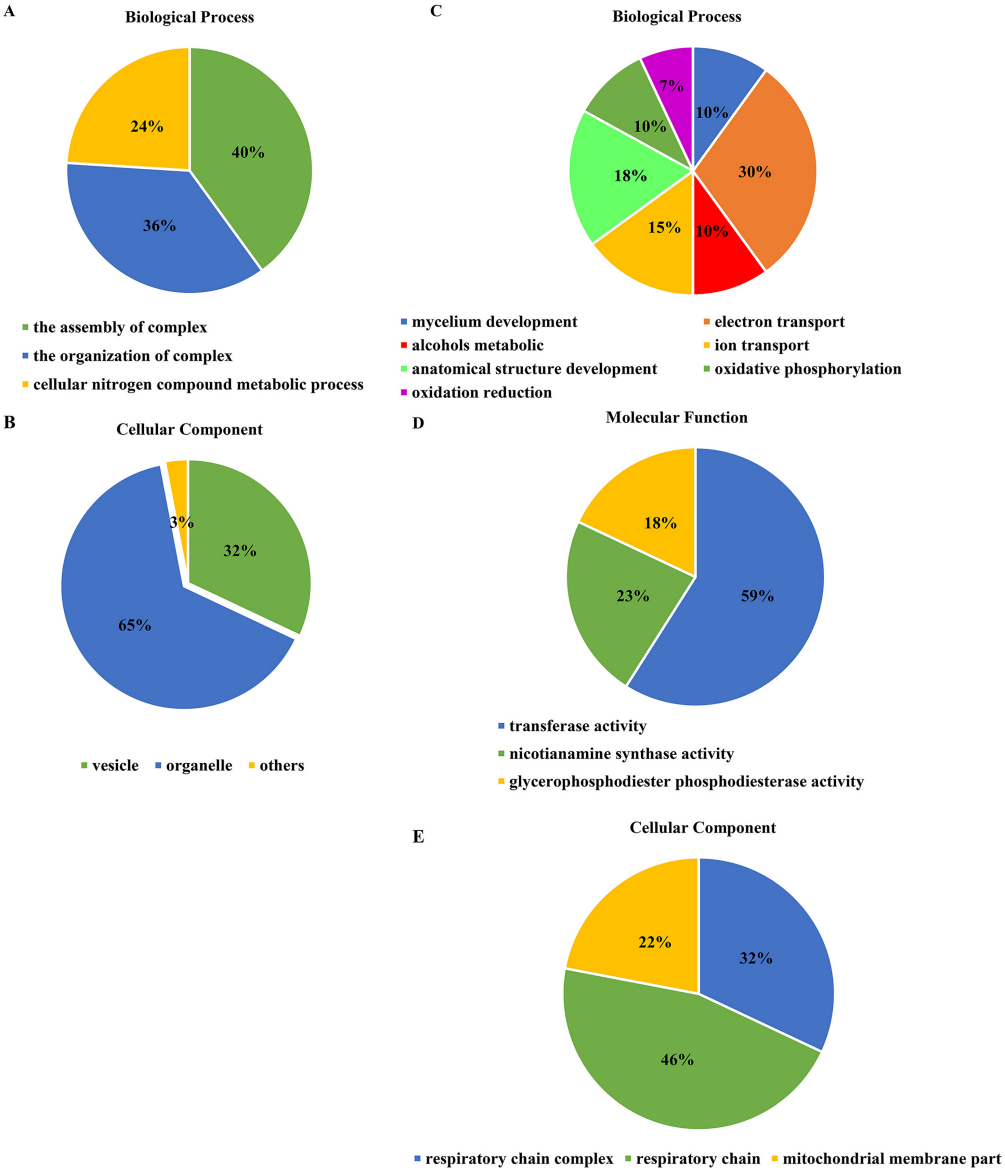
Effects of two phosphorus levels on functional category distribution of differentially expressed genes in Xindong20 and Xindong23 under drought stress. (A and B) Biological process and cellular component in Xindong20; (C–E) biological process, cellular component and molecular function in Xindong23.

For the DEGs in Xindong20, in the biological process category, about 40% of these genes were associated with the assembly of complex (including DNA packaging, nucleosome assembly, chromatin assembly, protein-DNA complex assembly, and chromatin assembly or disassembly), about 36% were associated with the organization of complex (including mycelium development, nucleosome organization, chromosome organization, and chromatin organization), and about 24% were associated with cellular nitrogen complex metabolic processes (Fig. 5A). The up-regulated genes accounted for 81% of the total in the biological process, but the genes associated with the mycelial development were down-regulated in Xindong20 (Table 3).

In the cellular component category, the DEGs mainly regulated the organelles and vesicles in Xindong20. Among them, about 65% and 32% were associated with organelle (70% was up-regulated) and vesicle (66% was up-regulated), respectively. Moreover, about 3% were associated with nucleosome, chromatin, chromosome, and protein-DNA complexes (Fig. 5B).

The number of DEGs in Xindong23 root was less than that in Xindong20 root under LP and CP at DS 7 d. In the biological process category, 30% of DEGs were associated with electron transport, 18% were associated with the development of root anatomical structure, 15% were associated with the ion transport, and 10% were associated with the alcohols material metabolism (Fig. 5C). Among them, five gene groups associated with electron transport and 14 genes associated with root anatomical structure development were all down-regulated, and 11 genes associated with root anatomical structure development were up-regulated (Table 4).

At DS 7 d, the molecular function of Xindong23 mainly included transferase activity (59%), nicotianamine synthase activity (23%), and glycerophosphodiester phosphodiesterase activity (22%) (Fig. 5D). Among them, all DEGs associated with nicotianamine synthase activity were down-regulated, and all genes associated with phosphodiesterase activity were up-regulated under LP compared with those under CP (Table 4).

In the cell component category, 46% of DEGs were associated with respiratory chain reaction, 32% were associated with respiratory chain complex, and 22% were associated with mitochondrial membrane part (Fig. 5E). Among them, 13 genes associated with respiratory chain complexes were all down-regulated (Table 4).

### Effects of two phosphorus levels on the element content of wheat roots under drought stress

**Figure 6:**
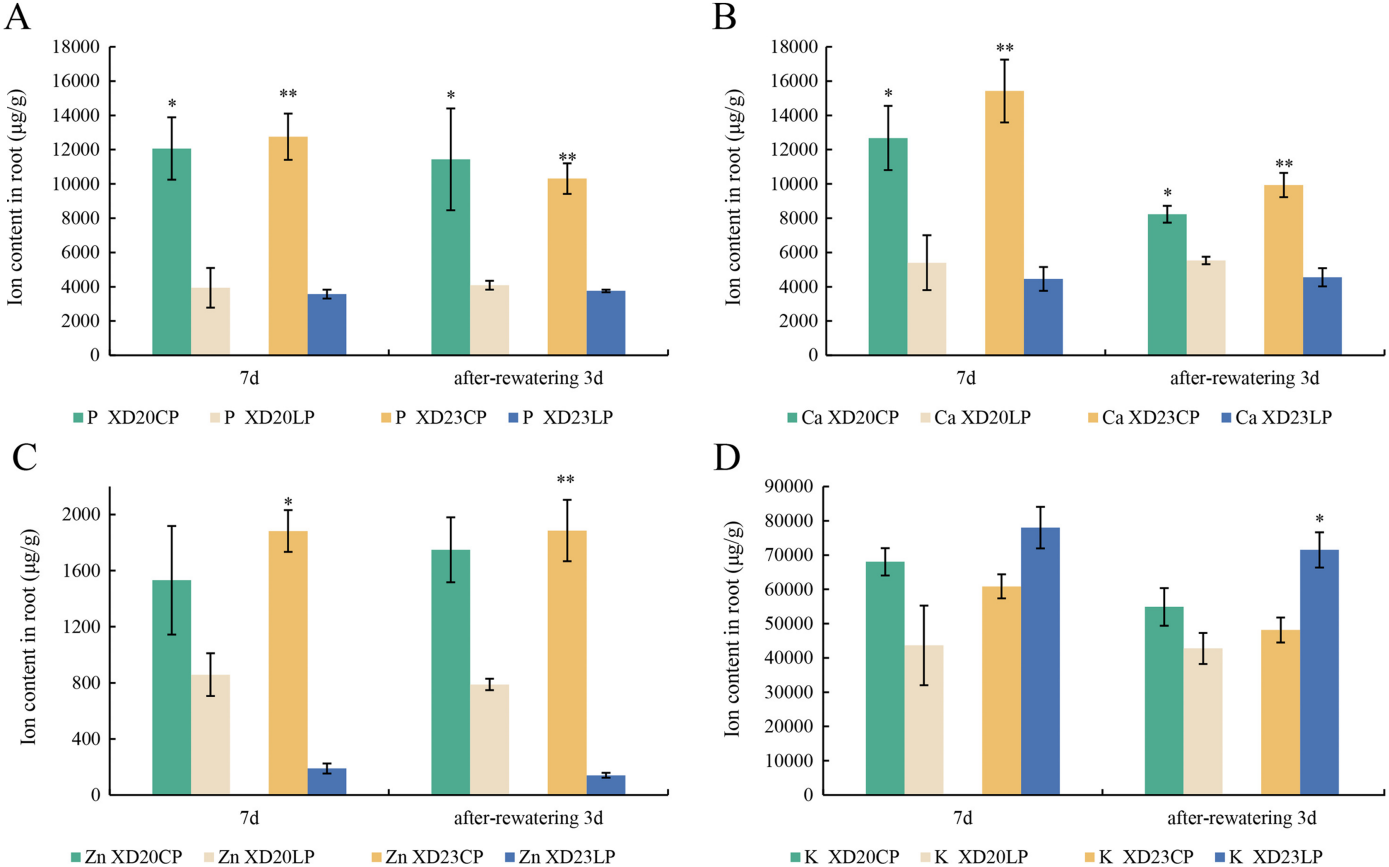
Effects of two phosphorus levels on the element content of wheat roots under drought stress. (A and B) Histogram of each element content in Xindong20 and Xindong23 roots. (C) Histogram of Zn element content in Xindong20 and Xindong23 roots. (D) Histogram of K element content in Xindong20 and Xindong23 roots. CP: 1.0 mmol/L; LP: 0.05 mmol/L; Values are means ± standard deviation of three repetitions; one asterisk (*) and two asterisks (**) are significantly different at *p* < 0.05 and *p* < 0.01, respectively. XD20CP: Xindong20 under conventional phosphorus level; XD20LP: Xindong20 under low phosphorus level; XD23CP: Xindong23 under conventional phosphorus level; XD23LP: Xindong23 under low phosphorus level.

The changes P and Ca content in two varieties were similar from DS 7 d to RW 3 d. The content of P and Ca were significantly higher under CP than under LP (Figs. 6A, 6B). The content of Zn was significantly lower under LP than under CP on all sampling days in Xindong23 (Fig. 6C). In addition, among the four nutrients studied, the content of K was the highest in the two varieties under LP and CP (Fig. 6D).

### qRT-PCR analysis

**Figure 7:**
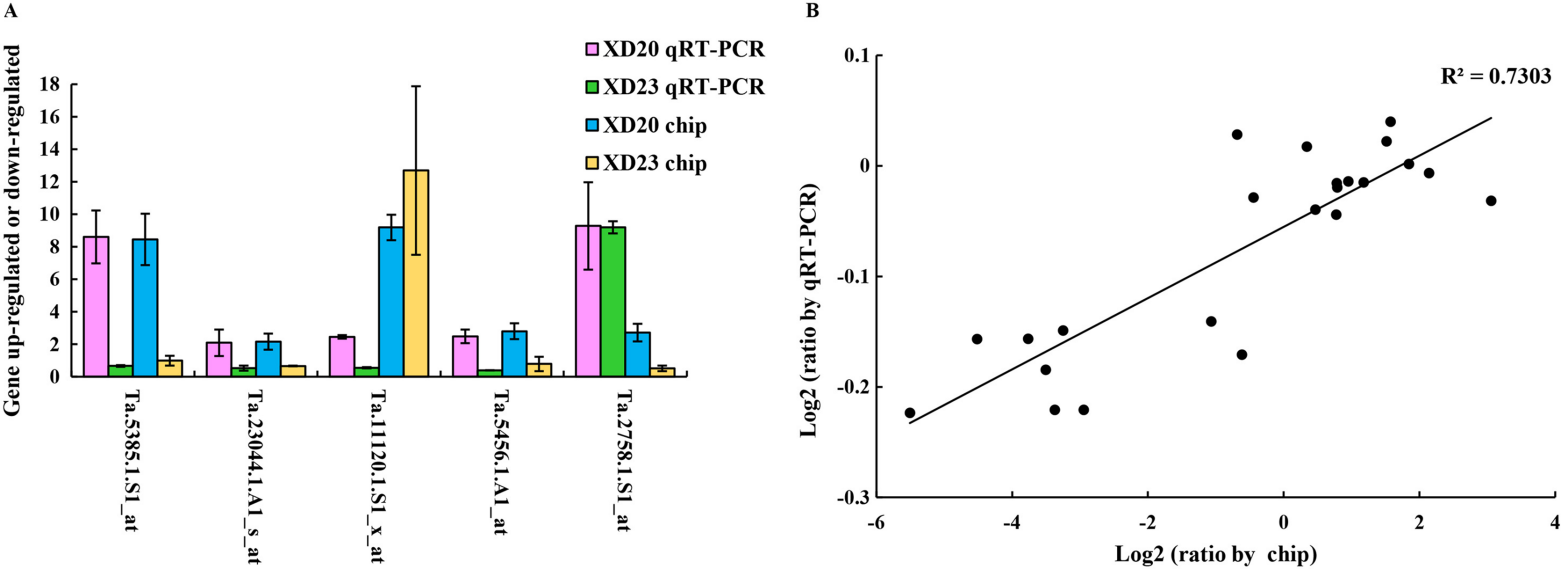
Validate gene chip results with qRT-PCR. (A) Five random genes were selected from XD20 and XD23. Bars represent the standard errors (mean ± SE) (*n* = 3). (B) Relationship between qRT-PCR and chip results of randomly selected genes. Value is the log_2_ ratio (LP/CP) of the gene. The correlation coefficient (R^2^) is shown in the figure. Three biological replicates for each PCR reaction.

Genes were randomly selected from two wheat varieties for qRT-PCR analysis to verify the accuracy and reliability of gene chip data. The results showed that the changes in gene expression in qRT-PCR and the chip were roughly similar, and the expression trend was very similar to the chip data (R^2^ = 0.73, *p* < 0.05) (Fig. 7), confirming that the results of the gene chip were accurate and reliable.

## Discussion

Root play a vital role in wheat responses to biotic and abiotic stresses. Previous studies showed that the absorption of P and water by plants were closely related to root morphology (Schiefelbein & Benfey, 1991; Osmont, Sibout & Hardtke, 2007). In this study, under drought stress, the root growth was better under LP than under CP in Xindong20. Under LP, the total P content in the roots of the two varieties was increased at RW 3 d compared with those at DS 7 d. Meanwhile, the decreases in root vitality and root absorption area induced by drought stress were suppressed under LP. Because P could promote the metabolism of plants, the rapid increase in P content could promote the recovery of plant physiological functions re-watering.

Drought stress not only suppresses the growth of wheat root, but also the growth of shoot (Liu & Stützel, 2004). In this study, under drought stress, the root water content of the two varieties were significantly lower under LP than under CP, but the shoot water content was on the contrary. This indicates that transport of water to wheat shoot could be promoted by LP under drought stress. At present, a variety of signal sensors have been used in the studies on how plant roots sense and respond to fluctuations in water and nutrients in the environment. Ca^2+^ is the common second messenger (Loro et al., 2016). Previous study showed that the response of Ca^2+^ *Arabidopsis* root tips to abiotic stress could be inhibited by P starvation (Matthus et al., 2019). In this study, under drought stress, the content of Ca in the roots of Xindong20 and Xindong23 was significantly lower under LP than under CP. Based on the above, in the arid regions, relatively low P application could promote the absorption of water and P by wheat root and stimulate wheat growth. However, the genotypic differences could not be ignored. The effect of low P supply on allevating drought stress was more evident in the P-sensitive wheat variety.

The microarray results showed that there were significant genotypic differences in the response to the two phosphorus levels under drought stress. The DEGs in Xindong20 (with strong P utilization efficiency and drought tolerance) were more than those in Xindong23. It indicates that more genes are regulated to improve the drought tolerance in Xindong20. This also confirms the characteristics of the two winter wheat varieties.

Plant P transporters play an important role in the uptake and redistribution of P (Brown et al., 2012). *OsPT1* is involved in the direct uptake of P from the soil, and *OsPT8* plays a role in redistributing P (Nagarajan et al., 2011; Sun et al., 2012; Li et al., 2015). In this study, *PT1* and *PT8* were up-regulated in Xindong20, and *PT8* was also up-regulated in Xindong23. This is consistent with the study results of the total P content of two varieties. In addition, K and P nutrition were closely linked (Kellermeier et al., 2014). Ródenas et al. (2017) found that in tomato and *Arabidopsis*, P deficiency could suppress the uptake of K and affect the expression of AKT1. Besides, the absorption of P by plants could be suppressed by high K^+^ content, resulting in the up-regulation of the gene associated with the root P absorption system (Ródenas et al., 2019). In this study, at DS 7 d, among the four nutrients studied, the content of K was the highest in two varieties under LP (Fig. 6D), and the content of P in the root were significantly reduced under LP. In addition, the increase of K content in Xindong23 root also confirmed the up-regulated expression of *PT8* (Fig. 6D). Under LP, the total P content of root was significantly higher than that of shoot in Xindong20 at DS 7 d. The total P content of root in Xindong23 at DS 7 d was significantly compared with that at DS 5 d (Fig. 2). This may be related to the adaptability of wheat to low P supply and the regulation of P. Previous studies found that the *WRKY*-type bound to the W-box in the *PHT1* promoters, and the expression of *PHT1* was regulated by *WRKY* (Wang et al., 2017). Zn and P, needed by plants to maintain their essential functions (Sinclair & Krämer, 2012), could regulate the growth of plants (Zhu, Smith & Smith, 2001). The microarray results showed that *WRKY19-b* was up-regulated in Xindong20 and down-regulated in Xindong23. It may indicate that *PT8* is down-regulated by *WRKY19-b*. This result is similar to the report of Devaiah, Karthikeyan & Raghothama (2007). In *Arabidopsis thaliana*, Zn starvation leads to the lowered expression of *PHT1;1* in root (Jain et al., 2013; Khan et al., 2014). Devaiah, Karthikeyan & Raghothama (2007) found that *AtPHT1;1* and *AtPHT1;4* were down-regulated when the expression of the *WRKY75* was down-regulated. Wang et al. (2014) and Ding et al. (2016) also obtained similar results. They found that the down-regulated expression of *WRKY45* could lead to the down-regulation of *AtPHT1;1*. Meanwhile, Dai, Wang & Zhang (2016) showed that the overexpression of *WRKY74* promoted the growth of rice rhizomes. In this study, the content of Zn in the roots of Xindong20 and Xindong23 were lower under LP than under CP at DS 7 d (Fig. 6C). Combined with the expression of *WRKY19-b* in two varieties (up-regulation in Xindong20 and down-regulation in Xindong23), maybe it could explain why the root growth of Xindong20 was better than that of Xindong23 under LP.

Silicon is important for improving the drought tolerance of wheat under drought stress (Gong et al., 2005). Agarie et al. (1998) showed that the membrane permeability and transpiration rate in rice were reduced by silicon application during polyethylene glycerol-induced water deficit. Gong et al. (2003) showed that silicon application could improve the drought tolerance and increase dry matter of wheat plants under drought stress compared to no silicon application. In this study, after re-watering, the root water content of Xindong20 was higher than that of Xindong23. Meanwhile, the microarray results showed that the gene associated with silicon transporterwas up-regulated in Xindong20 and down-regulated in Xindong23. Liang et al. (2003) found that silicon application could enhance the activity of superoxide dismutase (SOD) in the leaves, as well as the activity of SOD and peroxidase (POD) in the roots of barley under salt stress, thereby protecting plant tissue from oxidative damage and improving antioxidant capacity. Gong et al. (2005) also found that silicon application could reduce oxidative damage in wheat and enhance drought tolerance. In this study, in Xindong20, the expression of the gene associated with POD activity was up-regulated. This is consistent with the characteristics of the Xindong20 (high drought tolerance and P utilization efficiency).

The dry matter accumulation of wheat greatly depends on the photosynthesis. Triose phosphate is an intermediate product of photosynthesis. Macgregor et al. (2008) found that sucrose phosphate synthase was a key enzyme in the synthesis of sucrose from propyl phosphate, and its activity was affected by drought stress. In this study, the gene group associated with sucrose phosphate synthase were up-regulated both in Xindong20 and Xindong23. The accumulation of sucrose or starch in plant leaves alters the distribution of dry matter in the root, causing the changes in root structure and growth (Hermans et al., 2006). In this study, at DS 7 d, the difference in root dry weight between LP and CP treatment was not significant within the same variety. However, the difference in Xindong23 was more significant than that of Xindong20. The indicates that the effect of the external environment (water and P) on dry matter accumulation might also depend on the genotype differences.

The glycolysis process is one of the important metabolic pathways for plant. Previous study found that under a low P condition, the ATP-dependent phosphoglycerate kinase and NAD-dependent P dehydrogenase in the glycolytic metabolic pathway were affected, so that the glycolytic pathway decomposed sugar by inducing the synthesis of inorganic pyrophosphate enzyme to provide energy for growth and improve P utilization efficiency. Besides, UDP-glucose pyrophosphatase was also affected by the low P condition, which made the conversion of sucrose dependent on the pyrophosphate pathway. Pyrophosphate was catalyzed by inorganic pyrophosphatase to P while releasing a large amount of energy for use (Ciereszko et al., 2001). This study obtained similar results. In this study, the expression of the gene associated with inorganic pyrophosphatase was up-regulated in Xindong20. This may be one of the reasons why the P utilization efficiency of Xindong20 is higher than that of Xindong23.

Bernardo et al. (2017) found that under drought stress, the biosynthesis of soluble sugar, permease, and fructan in plants were increased to maintain normal growth. *6-SFT* (*fructan 6-fructosyltransferase*) is a key enzyme involved in fructan biosynthesis and the central reserve of carbohydrates. It has an osmotic protective effect on cell membranes under drought stress conditions (Livingston, Hincha & Heyer, 2009). In this study, the expression of the gene associated with sucrose: *6-SFT* was up-regulated in Xindong20, which could improve the biosynthesis of fructan. This could explain why Xindong20 has higher drought tolerance than Xindong23 to some extent.

Trehalose is involved in the responses to many abiotic stresses in plants. *Trehalose-6-phosphate synthase* (*TPS*) could affect the biosynthesis of trehalose for it plays a crucial role in the metabolism of glycolysis. In *Arabidopsis*, trehalose plays a critical role in the regulation of glucose and ABA signaling (Avonce et al., 2004). Iordachescu & Imai (2008) demonstrated that the plant tolerance to abiotic stresses was enhanced by the accumulation of trehalose. In this study, the down-regulation of *trehalose-6-phosphate synthase* in Xindong23 may lead to the repression of trehalose biosynthesis. This might be related to the poor drought tolerance of Xindong23.

In higher plants, ribonuclease plays an important role in degrading nucleic acid to release P. D’Alessio & Riordan (1997) found that the gene associated with ribonuclease degrades was up-regulated in roots under P starvation. In this study, the chip data showed that the gene expression associated with ribonuclease was up-regulated in Xindong23. This might be due to the deterioration of nucleic acid degradation in the cell of Xindong23 under drought stress. Under drought stress, the total P content in Xindong23 continuously decreased under LP. This indicates that drought stress may suppress the uptake of P by winter wheat.

## Conclusions

This study revealed the mechanism of wheat responses to low and conventional P levels under drought stress by a complex regulatory network. Under drought stress, the nutrient uptake by wheat root could be improved to some extent by the adjustment of physiological indexes, ion, and gene expression under a low P condition. This could reduce the effects of drought stress on wheat growth. Besides, there was certain genotypic differences in this effect. Xindong20 showed a better root growth in response to drought stress compared with Xindong23 under LP and CP. The genes associated with silicon transporters, phosphate transporters, sucrose synthesis, glycolysis process, carbohydrates synthesis, *etc*., were mostly up-regulated in Xindong20, while the genes associated with the electron transport chain and the respiratory chain, glycolysis process and degrading nucleic acids were mostly down-regulated in Xindong23. Therefore, under combined drought and P stresses, maintaining the cell structural integrity and reducing the energy metabolism in wheat roots which will help to improve the drought tolerance of wheat. In future study, we will explore the effects of low phosphorus treatment on wheat root growth at different concentrations of PEG-6000 (0%, 10%, 15%, and 25%) and the key genes in wheat responses, to further explain the effects of low phosphorus treatment on the growth of wheat under different levels of drought stress.

## Abbreviations

**P** Phosphorus

**Ca** Calcium

**K** Potassium

**Zn** Zinc

**DEGs** Differentially expressed genes

**CP** Conventional–phosphorus level

**LP** Low-phosphorus level

**FDR** False discovery rate

**GO** Gene Ontology

**SOD** Superoxide dismutase

**POD** Peroxidase

**6-SFT** Fructan 6-fructosyltransferase

**TPS** Trehalose-6-phosphate synthase

## Supplemental Information

10.7717/peerj.13887/supp-1 Supplemental Information 1 Completed MIAME checklist of GeneChip database. A completed MIAME checklist for Gene expression data of winter wheat root under drought stress for 7 days by two phosphorus treatments.

## Additional Information and Declarations

### Microarray Data Deposition

The following information was supplied regarding the deposition of microarray data: Data is available at the NCBI GEO database: GSE189698.

### Data Availability

The following information was supplied regarding data availability: The GeneChip data is available at NCBI GEO: GSE189698. The physiological data is available at figshare: Zhang, Xiangchi; Lu, Weidan; Wang, Xiaoli; Ma, Bin; Fu, Kaiyong; Li, Chunyan; et al. (2021): Data of physiological. figshare. Dataset. https://doi.org/10.6084/m9.figshare.17085479.v2. The morphological data is available at figshare: Zhang, Xiangchi; Lu, Weidan; Wang, Xiaoli; Ma, Bin; Fu, Kaiyong; Li, Chunyan; et al. (2021): Data of morphological characteristics. figshare. Dataset. https://doi.org/10.6084/m9.figshare.17075117.v2. The element content data is available at figshare: Zhang, Xiangchi; Lu, Weidan; Wang, Xiaoli; Ma, Bin; Fu, Kaiyong; Li, Chunyan; et al. (2021): Element content data of winter wheat root under drought stress by two phosphorus treatments. figshare. Dataset. https://doi.org/10.6084/m9.figshare.17091116.v1. The gene expression (qRT-PCR) data is available at figshare: Zhang, Xiangchi; Lu, Weidan; Wang, Xiaoli; Ma, Bin; Fu, Kaiyong; Li, Chunyan; et al. (2021): Gene Expression Data. figshare. Dataset. https://doi.org/10.6084/m9.figshare.17085485.v1. The raw scanner image of GeneChip is available at figshare: Zhang, Xiangchi; Lu, Weidan; Wang, Xiaoli; Ma, Bin; Fu, Kaiyong; Li, Chunyan; et al. (2021): Raw scanner image of GeneChip. figshare. Figure. https://doi.org/10.6084/m9.figshare.17091149.v1.

## References

[ref-1] Adams RP, Kendall EJ, Kartha KK (1990). Comparison of free sugars in growing and desiccated plants of *Selaginella lepidophylla*. Biochemical Systematics and Ecology.

[ref-2] Agarie S, Hanaoka N, Ueno O, Miyazaki A, Kubota F, Agata W, Kaufman PB (1998). Effects of silicon on tolerance to water deficit and heat stress in rice plants (*Oryza sativa* L.), monitored by electrolyte leakage. Plant Production Science.

[ref-3] Anjum SA, Xie X-Y, Wang L, Saleem MF, Man C, Lei W (2011). Morphological, physiological and biochemical responses of plants to drought stress. African Journal of Agricutural Research.

[ref-4] Avonce N, Leyman B, Mascorro-Gallardo JO, Van DP, Thevelein JM, Iturriaga G (2004). The *Arabidopsis* Trehalose-6-P Synthase *AtTPS1* gene is a regulator of glucose, abscisic acid, and stress signaling. Plant Physiology.

[ref-5] Bao SD (2000). Soil and agricultural chemistry analysis.

[ref-6] Behera S, Zhaolong X, Luoni L, Bonza MC, Doccula FG, De Michelis MI, Morris RJ, Schwarzländer M, Costa A (2018). Cellular Ca^2+^ signals generate defined pH signatures in plants. Plant Cell.

[ref-7] Bernardo L, Morcia C, Carletti P, Ghizzoni R, Badeck FW, Rizza F, Lucini L, Terzi V (2017). Proteomic insight into the mitigation of wheat root drought stress by arbuscular mycorrhizae. Journal of Proteomics.

[ref-8] Brown LK, George TS, Thompson JA, Wright G, Lyon J, Dupuy L, Hubbard SF, White PJ (2012). What are the implications of variation in root hair length on tolerance to phosphorus deficiency in combination with water stress in barley (*Hordeum vulgare*)?. Annals of Botany.

[ref-9] Ciereszko I, Johansson H, Hurry V, Kleczkowski LA (2001). Phosphate status affects the gene expression, protein content and enzymatic activity of UDP-glucose pyrophosphorylase in wild-type and *pho* mutants of Arabidopsis. Planta.

[ref-10] Condon AG, Richards RA, Rebetzke GJ, Farquhar GD (2004). Breeding for high water-use efficiency. Journal of Experimental Botany.

[ref-11] Cruz de Carvalho MH (2008). Drought stress and reactive oxygen species, production, scavenging and signaling. Plant Signaling & Behavior.

[ref-12] Dai X, Wang Y, Zhang WH (2016). *OsWRKY74*, a WRKY transcription factor, modulates tolerance to phosphate starvation in rice. Journal of Experimental Botany.

[ref-13] Deak KI, Malamy J (2005). Osmotic regulation of root system architecture. The Plant Journal.

[ref-14] Devaiah BN, Karthikeyan AS, Raghothama KG (2007). *WRKY75* transcription factor is a modulator of phosphate acquisition and root development in Arabidopsis. Plant Physiology.

[ref-15] Ding W, Wang Y, Fang W, Gao S, Li X, Xiao K (2016). *TaZAT8*, a C2H2-ZFP type transcription factor gene in wheat, plays critical roles in mediating tolerance to Pi deprivation through regulating P acquisition, ROS homeostasis, and root system establishment. Physiologia Plantarum.

[ref-16] D’Alessio G, Riordan JF (1997). Ribonucleases: structures and functions.

[ref-17] Epstein E (1999). Silicon. Annual Review of Plant Physiology and Plant Molecular Biology.

[ref-18] Gao JF (2006). Experimental guidance for pant physiology.

[ref-19] Garcia K, Delteil A, Conejero G, Becquer A, Plassard C, Sentenac H, Zimmermann S (2014). Potassium nutrition of ectomycorrhizal Pinus pinaster: overexpression of the Hebeloma cylindrosporum HcTrk1transporter affects the translocation of both K(+) and phosphorus in the host plant. New Phytologist.

[ref-61] Gong HJ, Chen KM, Chen GC, Wang SM, Zhang CL (2003). Effects of silicon on growth of wheat under drought. Journal of Plant Nutrition.

[ref-20] Gong HJ, Zhu XY, Chen KM, Wang SM, Zhang CL (2005). Silicon alleviates oxidative damage of wheat plants in pots under drought. Plant Science.

[ref-21] Harrison MJ, Dewbre GR, Liu J (2002). A phosphate transporter from *Medicago truncatula* involved in the acquisition of phosphate released by arbuscular mycorrhizal fungi. The Plant Cell.

[ref-22] Hermans C, Hammond JP, White PJ, Verbruggen N (2006). How do plants respond to nutrient shortage by biomass allocation?. Trends in Plant Science.

[ref-59] Hurd EA (1975). Phenotype and drought tolerance in wheat. Developments in Agricultural and Managed Forest Ecology.

[ref-23] Iordachescu M, Imai R (2008). Trehalose biosynthesis in response to abiotic stresses. Journal of Integrative Plant Biology.

[ref-24] Jain A, Sinilal B, Dhandapani G, Meagher RB, Sahi SV (2013). Effects of deficiency and excess of zinc on morphophysiological traits and spatiotemporal regulation of zinc-responsive genes reveal incidence of cross talk between micro- and macronutrients. Environmental Science Technology.

[ref-25] Kang HM, Saltveit ME (2002). Reduced chilling tolerance in elongating cucumber seedling radicles is related to their reduced antioxidant enzyme and DPPH-radical scavenging activity. Physiologia Plantarum.

[ref-26] Katrin WF, Ralf U, Verena P, Klaus JD, Silvia BP, Mareike Kavka (2019). Genotype-Specific Differences in Phosphorus Efficiency of Potato (*Solanum tuberosum* L.). Frontiers in Plant Science.

[ref-27] Kellermeier F, Armengaud P, Seditas TJ, Danku J, Salt DE, Amtmann A (2014). Analysis of the root system architecture of arabidopsis provides a quantitative readout of crosstalk between nutritional signals. Plant Cell.

[ref-28] Khan GA, Bouraine S, Wege S, Li Y, de Carbonnel M, Berthomieu P, Poirier Y, Rouached H (2014). Coordination between zinc and phosphate homeostasis involves the transcription factor PHR1, the phosphate exporter PHO1, and its homologue PHO1;3 in Arabidopsis. Journal of Experimental Botany.

[ref-29] Li YT, Zhang J, Zhang X, Fan HM, Gu HY, Xu GH (2015). Phosphate transporter *OsPht1;8* in rice plays an important role in phosphorus redistribution from source to sink organs and allocation between embryo and endosperm of seeds. Plant Science.

[ref-30] Liang Y, Chen Q, Liu Q, Zhang W, Ding R (2003). Exogenous silicon (Si) increases antioxidant enzyme activity and reduces lipid peroxidation in roots of salt-stressed barley (*Hordeum vulgare* L.). Journal of plant physiology.

[ref-31] Liu F, Stützel H (2004). Biomass partitioning, specific leaf area, and water use efficiency of vegetable amaranth (*Amaranthus spp*.) in response to drought stress. Scientia Horticulturae.

[ref-32] Liu F, Wang Z, Ren H, Shen C, Ling HQ, Wu CY, Lian XM, Ping W (2010). *OsSPX1* suppresses the function of *OsPHR2* in the regulation of expression of *OsPT2* and phosphate homeostasis in shoots of rice. The Plant Journal.

[ref-33] Livingston DP, Hincha DK, Heyer AG (2009). Fructan and its relationship to abiotic stress tolerance in plants. Cellular and Molecular Life Sciences.

[ref-34] Loro G, Wagner S, Doccula FG, Behera S, Weinl S, Kudla J, Schwarzländer M, Costa A, Zottini M (2016). Chloroplast-specific in vivo Ca^2+^ imaging using Yellow Cameleon fluorescent protein sensors reveals organelle-autonomous Ca^2+^ signatures in the stroma. Plant Physiology.

[ref-35] Macgregor DR, Deak KI, Ingram PA, Malamy JE (2008). Root system architecture in *Arabidopsis* grown in culture is regulated by sucrose uptake in the aerial tissues. The Plant Cell.

[ref-36] Manishankar P, Wang N, Köster P, Alatar AA, Kudla J (2018). Calcium signaling during salt stress and in the regulation of ion homeostasis. Journal of Experimental Botany.

[ref-37] Manske GGB, Ortiz-Monasterio JI, van Ginkel M, Gonzalez RM, Fischer RA, Rajaram S, Vlek PLG (2001). Importance of P uptake efficiency versus P utilization for wheat yield in acid and calcareous soils in Mexico. European Journal of Agronomy.

[ref-38] Matthus E, Wilkins KA, Swarbreck SM, Doddrell NH, Doccula FG, Costa A, Davies JM (2019). Phosphate starvation alters abiotic-stress-induced cytosolic free calcium increases in roots. Plant Physiology.

[ref-39] Moller IM, Jensen PE, Hansson A (2007). Oxidative modifications to cellular components in plants. Annual Review of Plant Biology.

[ref-40] Nagarajan VK, Jain A, Poling MD, Lewis AJ, Raghothama KG, Smith AP (2011). *Arabidopsis Pht1;5* mobilizes phosphate between source and sink organs and influences the interaction between phosphate homeostasis and ethylene signaling. Plant Physiology.

[ref-41] Nussaume L, Kanno S, Javot H, Marin E, Pochon N, Ayadi A, Nakanishi TM, Thibaud MC (2011). Phosphate import in plants: focus on the PHT1 transporters. Frontiers in Plant Science.

[ref-42] Osmont KS, Sibout R, Hardtke CS (2007). Hidden branches: developments in root system architecture. Annual Review of Plant Biology.

[ref-43] Paszkowski U, Kroken S, Roux C, Briggs SP (2002). Rice phosphate transporters include an evolutionarily divergent gene specifically activated in arbuscular mycorrhizal symbiosis. Proceedings of the National Academy of Sciences of the United States of America.

[ref-44] Ródenas R, García-Legaz MF, López-Gómez E, Martínez V, Rubio F, Ángeles Botella M (2017). NO_3_^−^, PO_4_^3−^ and SO_4_^2−^ deprivation reduced LKT1-mediated low-affinity K^+^ uptake and SKOR-mediated K^+^ translocation in tomato and Arabidopsis plants. Physiology Plant.

[ref-45] Ródenas R, Martínez V, Nieves-Cordones M, Rubio F (2019). High External K^+^ concentrations Impair Pi nutrition, induce the phosphate starvation response, and reduce arsenic toxicity in arabidopsis plants. International Journal of Molecular Sciences.

[ref-46] Savant NK, Korndörfer GH, Datnoff LE, Snyder GH (1999). Silicon nutrition and sugarcane production: a review. Journal of Plant Nutrition.

[ref-47] Schiefelbein JW, Benfey PN (1991). The development of plant root: new approaches to underground problem. The Plant Cell.

[ref-48] Sharma P, Dubey RS (2005). Drought induces oxidative stress and enhances the activities of antioxidant enzymes in growing rice seedlings. Plant Growth Regulation.

[ref-49] Sinclair SA, Krämer U (2012). The zinc homeostasis network of land plants. Biochimica et Biophysica Acta.

[ref-50] Sun S, Gu M, Cao Y, Huang X, Zhang X, Ai P, Zhao JN, Fan XR, Xu GH (2012). A constitutive expressed phosphate transporter, *OsPht1;1*, modulates phosphate uptake and translocation in phosphate-replete rice. Plant Physiology.

[ref-51] Türkan I, Bor M, Özdemir F, Koca H (2005). Differential responses of lipid peroxidation and antioxidants in the leaves of drought-tolerant *P. acutifolius* Gray and drought-sensitive *P. vulgaris* L. subjected to polyethylene glycol mediated water stress. Plant Science.

[ref-52] Vance CP, Uhde-Stone C, Allan DL (2003). Phosphorus acquisition and use: critical adaptations by plants for securing a nonrenewable resource. New Phytologist.

[ref-53] Wang F, He Z, Sayre K, Li SD, Si JS, Bo F, Kong LG (2009). Wheat cropping systems and technologies in China. Field Crops Research.

[ref-54] Wang D, Lv S, Jing P, Li Y (2017). Roles, regulation, and agricultural application of plant phosphate transporters. Frontiers in Plant Science.

[ref-55] Wang H, Xu Q, Kong YH, Chen Y, Duan JY, Wu WH, Chen YF (2014). *Arabidopsis WRKY45* transcription factor activates *PHOSPHATE TRANSPORTER1;1* expression in response to phosphate starvation. Plant Physiology.

[ref-60] Wu ZZ, Ying YQ, Zhang YB, Bi YF, Wang AK, Du XH (2018). Alleviation of drought stress in Phyllostachys edulis by N and P application. Scientific Reports.

[ref-56] Zhang XZ (1990). Research methods of crop physiology.

[ref-57] Zhou Z, Wang Z, Lv Q, Shi J, Zhong Y, Wu P, Ma CZ (2015). SPX proteins regulate Pi homeostasis and signaling in different subcellular level. Plant Signaling & Behavior.

[ref-58] Zhu YG, Smith SE, Smith FA (2001). Plant growth and cation composition of two cultivars of spring wheat (*Triticum aestivum* L.) differing in P uptake efficiency. Journal of Experimental Botany.

